# Neighborhood Characteristics and Accelerated Aging: Evidence From the Health and Retirement Study

**DOI:** 10.1093/geroni/igab046.944

**Published:** 2021-12-17

**Authors:** Haena Lee, Jennifer Ailshire, Eileen Crimmins

**Affiliations:** University of Southern California, Los Angeles, California, United States

## Abstract

An individual’s rate of aging directly impacts one’s functioning, morbidity and mortality. Identifying factors related to accelerated or delayed aging may provide important information for potential areas of intervention. While race/ethnicity, socioeconomic status and behavior characteristics have been linked to biological aging, it is unclear whether neighborhood characteristics are associated with one’s rate of aging. We use a novel aging measure, Expanded Biological Age, from the 2016 Health and Retirement Study Venous Blood Study (HRS-VBS) to investigate whether individuals living with unfavorable neighborhood conditions are experiencing accelerated aging compared to those living in more favorable conditions. We constructed a summary measure of expanded biological age using 22 novel biomarkers in the HRS-VBS; we then regressed the summary measure on age and used the residuals as indicators of accelerated or delayed aging. We measured neighborhood physical disorder, presence of green space, and perceived social cohesion using the 2016 HRS Interviewer Observation data and Self-Administered Questionnaire. We find that individuals living with higher levels of neighborhood physical disorder appeared 1.05 years older biologically than the average for those of the same chronological age. Individuals living near green space including parks were 1.5 years younger biologically than expected based on their chronological age though this association was marginally significant. We did not find an association between neighborhood social cohesion and accelerated aging. This implies that living with severe neighborhood disorder, characterized by presence of disrepair, trash/litter, and abandoned structures, and living near green space, play an important role in who lives longer.

